# Supplementary material for: Individual retrotransposon integrants are differentially controlled by KZFP/KAP1-dependent histone methylation, DNA methylation and TET-mediated hydroxymethylation in naïve embryonic stem cells
Source: Epigenetics Chromatin. 2018 Feb 26;11:7. doi: 10.1186/s13072-018-0177-1 (PMC6389204; doi:10.1186/s13072-018-0177-1)
Supplement: Supplementary file 11 — Additional file 11. Pattern analysis. [file 13072_2018_177_MOESM11_ESM.zip › Patterns analysis/DataTables/extensions/Responsive/examples/initialisation/index.html]

Responsive examples - Initialisation


# Responsive example Initialisation

Responsive can be run on a DataTable in a number of different ways:

- By adding the class `responsive` or `dt-responsive` to the `table`
- Using the `responsiveR` option in the DataTables
  initialisation
- Use the `$.fn.dataTable.Responsive` constructor.

This set of examples demonstrates these initialisation options.

### Basic initialisation

- Class name
- Configuration option
- `new` constructor
- Ajax data
- Default initialisation

Please refer to the DataTables documentation for full
information about its API properties and methods.  
Additionally, there are a wide range of extras and
plug-ins which extend the capabilities of
DataTables.

DataTables designed and created by SpryMedia Ltd © 2007-2014  
DataTables is licensed under the MIT license.
